# Nocturnal Cerebral Oxygenation in Low‐Altitude Residents With Pulmonary Vascular Disease Staying Overnight at High Altitude. Data From a Randomized, Controlled, Crossover Trial

**DOI:** 10.1002/pul2.70363

**Published:** 2026-07-17

**Authors:** Carolin Kränzle, Vera Bissig, Simon R. Schneider, Julian Müller, Kay von Gruenigen, Meret Bauer, Laura Mayer, Lea Lüönd, Tanja Ulrich, Aglaia Forrer, Arcangelo Carta, Ester I. Schwarz, Konrad E. Bloch, Mona Lichtblau, Michael Furian, Silvia Ulrich

**Affiliations:** ^1^ Clinic of Pulmonology University Hospital Zurich Zurich Switzerland

## Abstract

Whether hypoxia during popular high‐altitude travel negatively affects cerebral oxygenation in vulnerable patients with pulmonary vascular disease (PVD) is unknown. We studied overnight cerebral tissue oxygen saturation (CTO) and desaturation index (cODI) in PVD‐patients at 2500 m and effects of supplemental oxygen therapy (SOT). In this randomized‐controlled crossover trial, stable PVD‐patients diagnosed with pulmonary arterial or distal chronic thromboembolic pulmonary hypertension (PAH/CTEPH) had CTO and cODI (decrease in CTO ≥ 4%) assessed overnight at 470 m and 2500 m along with fingertip pulseoximetry (SpO_2_) and desaturation index (aODI). For safety, SOT was given if SpO_2_ dropped < 80% for > 30 min and stratified analysis accordingly. Primary endpoint was the difference in CTO between 470 and 2500 m. 16 PVD‐patients (7 women; 13 PAH, 3 CTEPH), (mean ± SD) 56 ± 14 years old, were included per‐protocol. At 470 m, the mean nocturnal CTO was 66.3 ± 1.6% and SpO_2_ 90.3 ± 0.8%. At 2500 m on ambient air, CTO was unchanged 65.9 ± 1.6% (mean difference −0.4%; 95% CI −4.0 to 3.2) despite a lower SpO_2_ of 83.6 ± 0.8; −6.7 (−8.7 to −4.6%). At 2500 vs. 470 m, cODI increased by 3.1 events/hour (−0.3 to 6.6). SOT needed by 50% of PH‐patients improved SpO_2_ by +7.5 (4.9 to 10.0), but not CTO and cODI (+3.6% (−1.0 to 8.2)) and −3.8 events/hour (−8.1 to 0.5). In PVD‐patients on ambient air at 2500 m, nocturnal CTO remained unchanged compared to 470 m despite lower SpO_2_. SOT promptly improved SpO_2_ without affecting cerebral oxygenation. These findings suggest sufficient nocturnal cerebral protection at 2500 m in PVD.

## 1 Introduction

High‐altitude travel has become increasingly popular for both leisure and professional purposes. While healthy individuals usually tolerate hypoxia at high altitude, patients with pulmonary hypertension (PH) due to pulmonary vascular diseases (PVD), including pulmonary arterial hypertension (PAH) or distal chronic thromboembolic pulmonary hypertension (CTEPH), may be particularly vulnerable. These patients are at increased risk for altitude‐induced hypoxemia and altitude‐related complications due to impaired pulmonary hemodynamics [1, 2]. Previous work in patients with PH traveling to the hypoxic environment at high altitude has focused on tolerability and need for supplemental oxygen therapy (SOT) based on severe hypoxemia that was assessed by peripheral SpO_2,_ pulmonary hemodynamics or exercise performance [3, 4, 5]. In contrast, little is known about cerebral oxygen homeostasis in this high‐risk group at high altitude. This is of particular importance, as the brain tissue is highly sensitive to hypoxia. Sleep represents a vulnerable physiological state, especially in patients with cardiopulmonary disease, as nocturnal hypoventilation, central sleep apnea and reduced autonomic and hemodynamic compensation may aggravate hypoxemia [6, 7]. Accordingly, nocturnal desaturation has been identified as a clinically relevant phenomenon in patients with chronic respiratory diseases, such as chronic obstructive pulmonary disease or PVD especially during altitude exposure [8, 9]. Therefore, this study aimed to examine nocturnal cerebral oxygenation and desaturation patterns in relation to peripheral arterial oxygenation in PVD patients at high altitude and to assess the effect of SOT.

## 2 Methods

### 2.1 Study Design, Participants and Study Setting

This randomized, controlled, crossover trial was conducted between October 2021 and April 2022 (ClinicalTrials.gov: NCT05107700). Included were adults of any gender with a diagnosis of PAH or distal CTEPH, either inoperable or persistent after pulmonary endarterectomy, according to prevailing guidelines [1]. All patients were in stable clinical condition, with no changes to PH‐specific therapy for at least 4 weeks prior to enrollment. Exclusion criteria included a resting arterial oxygen tension (PaO_2_) < 8 kPa at low altitude, recent exposure to altitudes above 1000 m for more than three consecutive nights within the past 4 weeks, inability to comply with study procedures, relevant comorbidities, pregnancy, or breastfeeding.

Patients were assessed at low altitude (470 m) in Zurich and at high altitude (2500 m) on Mount Saentis in Switzerland during a 30‐h stay, including the first night at high altitude. The present investigation is part of a larger randomized, controlled crossover study assessing the effects of acute exposure to high altitude in patients with PVD [3, 4, 10]. While the overall study protocol included multiple physiological assessments, the current analysis specifically focuses on nocturnal measurements obtained during a night at 470 m and the first at 2500 m, which have not been published previously. Continuous recordings were analyzed between 10:00 p.m. and 6:00 a.m., corresponding to the predefined nocturnal observation period. The study followed a randomized‐sequence crossover design, with patients assigned to two randomly selected orders (low altitude – high altitude vs. high altitude – low altitude) and a washout period of more than 2 weeks between study sites. Transportation from the patients' homes to 2500 m was completed within 3 h by car and ropeway.

### 2.2 Assessments and Derived Parameters

#### 2.2.1 Near‐Infrared Spectroscopy

Cerebral tissue oxygen saturation (CTO) as primary outcome was assessed using near‐infrared spectroscopy (NIRS). Bilateral sensors were placed high on the forehead to continuously record oxygenated hemoglobin (O_2_Hb) and deoxygenated hemoglobin (HHb).

This technique measures concentration changes of O_2_Hb and HHb within the sampled cortical brain tissue based on differential light absorption at multiple wavelengths. Total hemoglobin (totHb) was calculated as the sum of O_2_Hb and HHb. CTO was calculated as the ratio of O_2_Hb to totHb and expressed as a percentage within the sampled cortical brain volume. In addition to CTO, the normalized tissue hemoglobin index (nTHI) was recorded as relative parameter derived from the NIRS signal and reflects relative changes in totHb concentration within the sampled tissue rather than absolute hemoglobin values. It is commonly interpreted as a surrogate marker of changes in cerebral blood volume and tissue perfusion over time [11].

### 2.3 Cerebral Oxygen Desaturation Indices

Cerebral oxygen desaturation events were defined as a decrease in CTO of at least 4% from baseline lasting for ≥ 10 s that occurred concurrently with a desaturation in SpO_2_ of at least 3% for ≥ 10 s. The cerebral oxygen desaturation index (cODI) was calculated as the number of cerebral desaturation events per hour. Signal preprocessing included removal of artefacts and exclusion of invalid data segments according to predefined criteria. In addition, severe cerebral oxygen desaturation events were analyzed separately. Severe cerebral desaturations were defined as CTO decreases of ≥ 13% from baseline lasting for ≥ 10 s, in line with thresholds used in previous NIRS studies at altitude [10]. The number of severe cerebral desaturation events was reported as absolute counts (*n*) and as events/hour. CTO and cODI were derived from NIRS signals using a customized MATLAB‐based analysis program. The methodological approach for calculation of CTO and cODI has been described previously and applied in studies investigating the effects of sleep, breathing disturbances, exercise, and altitude exposure on cerebral oxygenation in healthy individuals and patients with respiratory disease [12].

### 2.4 Cardiorespiratory Polygraphy and Arterial Oxygen Desaturation

Peripheral arterial oxygen saturation (SpO_2_) was recorded continuously by pulse oximetry. Arterial oxygen desaturation events (aODI) were defined as decreases in SpO_2_ of at least 3% lasting for ≥ 10 s. The aODI was calculated as the number of arterial desaturation events per hour.

### 2.5 Clinical Parameters and Supplemental Oxygen Therapy

Clinical parameters, including systolic and diastolic blood pressure and heart rate, were assessed at both altitudes. Patients were monitored continuously throughout the nocturnal observation period. SOT was administered if predefined safety criteria were met, defined as SpO_2_ < 80% for more than 30 min [13]. Night‐time on ambient air at 2500 m and with SOT were analyzed separately.

### 2.6 Outcomes

The primary outcome of this study was the difference in CTO during the first night at each altitude on ambient air. Secondary outcomes included cerebral and arterial oxygen desaturation indices (cODI and aODI), SpO_2_, other NIRS‐derived hemoglobin parameters, as well as clinical parameters including blood pressure and heart rate.

### 2.7 Statistical Analysis

Patients completing the study were included in the final per‐protocol analysis. Mixed linear regression models were employed to examine relationships between dependent variables (e.g., SpO_2_, CTO, and desaturation indices) and independent variables, such as low altitude and high altitude, and intervention (ambient air vs. SOT). Individual participant identifiers were included as random effects to account for repeated measures. Results are presented as means ± standard deviation or numbers (percentages), with 95% confidence intervals (CIs). To address specific secondary hypotheses, additional mixed linear regression models evaluated the relationship between mean CTO and factors such as mean SpO_2_, aODI, age and altitude. Statistical analyses were performed using R software. Statistical significance was defined as a two‐tailed *p*‐value < 0.05.

## 3 Results

**Figure 1:**
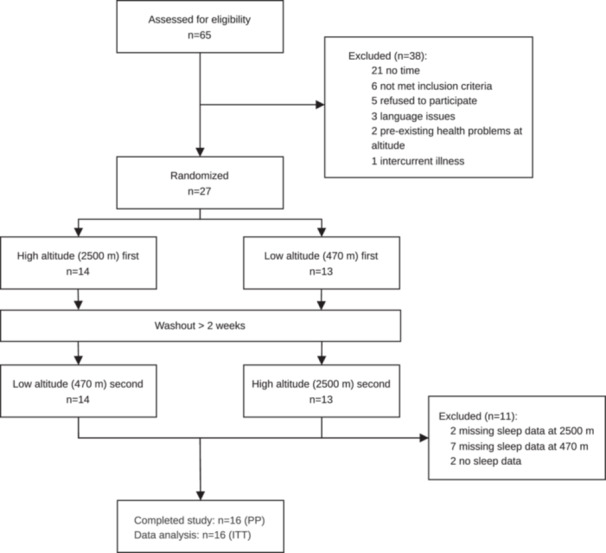
Study flowchart. ITT, intention to treat; PP, per protocol.

The patient flow is illustrated in Figure 1. Of 65 patients screened for eligibility, 27 were randomized, and 16 (44% women) were included in this analysis with a mean age of 56 ± 14 years and a mean BMI of 24.0 ± 3.7 kg/m^2^. Thirteen patients (81%) had PAH (62.5% idiopathic PAH, 18.5% PAH associated with other conditions), and three patients (19%) had distal CTEPH. According to the NYHA classification, 43.7% were in Class I, 43.7% in Class II, 6.3% in Class III, and one patient was unclassified. At the last routine pulmonary evaluation prior to study enrollment, mean arterial PaO_2_ was 10.2 ± 1.8 kPa (Table 1).

**Table 1:** Baseline characteristics at low altitude.

*n*, % women	16 (44%)
Age, years	56 ± 14
Body‐mass‐index, kg/m^2^	24.0 ± 3.7
Pulmonary arterial hypertension	13 (81%)
– Idiopathic	10 (62.5% of all patients)
– Associated with other condition	3 (18.5% of all patients)
Distal chronic thromboembolic pulmonary hypertension	3 (19%)
PAH‐specific medication	12 (75%)
– PDE‐5‐Inhibitors/soluble guanylate cyclase	10 (83%)
– Endothelin receptor antagonist	11 (92%)
– Prostanoids	5 (42%)
NYHA Class 1,2,3, unknown	7 (43.7%), 7 (43.7%), 1 (6.3%), 1 (6.3%)
6‐min walking distance, m	610 ± 88
Maximal cycle work capacity, Watts	142 ± 46
Peak oxygen uptake, ml/kg/min	20.5 ± 5.4
Mean pulmonary artery pressure, mmHg	46 ± 13
Pulmonary vascular resistance, WU	6.6 ± 3.1
Arterial partial pressure of oxygen, kPa	10.2 ± 1.8
Diffusion lung capacity, % predicted	64 ± 12

*Note:* Data are presented as mean ± SD or number (%).

Abbreviation: NYHA, New York Heart Association Classification.

### 3.1 Altitude Effect on Arterial and Cerebral Oxygenation

**Figure 2:**
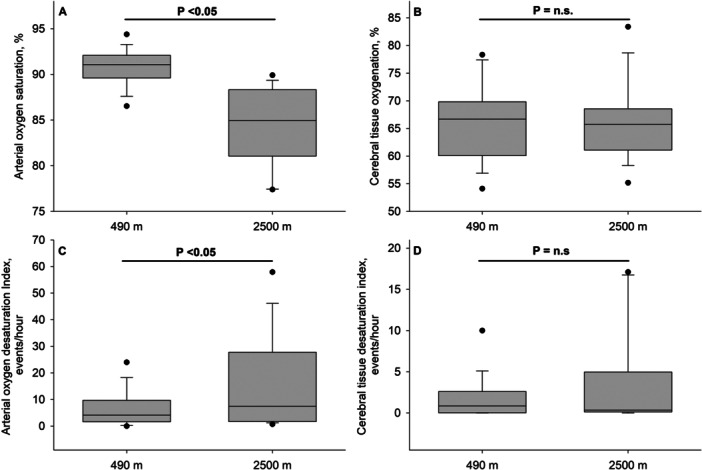
Box plot of mean arterial oxygen saturation (SpO_2_) [%] (Panel A), cerebral tissue oxygenation [%] (Panel B), arterial oxygen desaturation index [events/hour] (Panel C), and cerebral tissue desaturation index [events/hour] (Panel D) compared between 470 m and 2500 m. The significance of each result is given with P = n.s. or *p* < 0.05.

The effects of high altitude on peripheral arterial and cerebral oxygenation are summarized in Table 2 and Figure 2. Regarding the primary outcome, mean CTO on ambient air did not differ between low and high altitude (66.3% ± 1.6% at 470 m vs. 65.9% ± 1.6% at 2500 m), with a mean altitude effect of −0.4 (95% CI −4.0 to 3.2). aODI showed an increase of 11.2 events/h (3.3 to 19.2) at altitude (6.6 ± 3.2 events/h at 470 m vs. 17.8 ± 3.2 events/h at 2500 m). cODI showed a mild but non‐significant increase of 3.1 (−0.3 to 6.6) with altitude. Similar trends were seen in duration, percentage and number of severe cODI desaturations ≥ 13% (Table 2). SpO_2_ decreased significantly from 90.3% ± 0.8% at 470 m to 83.6% ± 0.8% at 2500 m, corresponding to a mean altitude effect of −6.7% (−8.7 to −4.6). This reduction in SpO_2_ was accompanied by a pronounced increase in aODI events. As shown in Figure 2, regression analyses revealed a significant altitude‐related decrease in mean nocturnal SpO_2_ and an increase in aODI, while mean CTO and cODI remained unchanged between 470 m and 2500 m.

**Table 2:** Overnight assessment at 470 and 2500 m on ambient air and effects of supplemental oxygen therapy (SOT) given to 8 patients with pulmonary vascular disease.

Variable	470 m	2500 m, ambient air	Mean altitude effect (95% CI)	2500 m, SOT	Mean SOT effect (95% CI)
*N*	16	16	16	8	8
Time in bed#, min	538 ± 39	331 ± 39	−207 (−313 to −100)	437 ± 108	105 (−25 to 236.0)
SpO_2_, %	90.3 ± 0.8	83.6 ± 0.8	−6.7 (−8.7 to −4.6)	91.1 ± 1.2	7.5 (4.9 to 10.0)
aODI, events/hour	6.6 ± 3.2	17.8 ± 3.2	11.2 (3.3 to 19.2)	1.6 ± 4.4	−16.2 (−26.2 to −6.3)
CTO, %	66.3 ± 1.6	65.9 ± 1.6	−0.4 (−4.0 to 3.2)	69.5 ± 2.1	3.6 (−1.0 to 8.2)
cODI, events/hour	1.6 ± 1.4	4.7 ± 1.4	3.1 (−0.3 to 6.6)	1 ± 1.9	−3.8 (−8.1 to 0.5)
cODI, duration, sec	16.6 ± 2.7	20.5 ± 2.5	3.9 (−3.3 to 11.1)	26.2 ± 3.4	5.6 (−2.5 to 13.8)
cODI, average, %	5.5 ± 2.8	8.4 ± 2.6	3.0 (−3.4 to 9.3)	12.0 ± 3.4	3.5 (−3.7 to 10.8)
cODI, ≥ 13%, *n*	1.1 ± 1.9	3.1 ± 1.9	2.0 (−3.1 to 7.1)	4.1 ± 2.7	1.0 (−5.3 to 7.3)
Systolic BP, mmHg	111 ± 2	110 ± 3	−0.3 (−6.1 to 5.5)	112 ± 2	1.6 (−4.2 to 7.4)
Diastolic BP, mmHg	65 ± 1	69 ± 2	4.0 (0.3 to 7.6)	69 ± 1	−1.3 (−5.0 to 2.4)
Heart rate, bpm	66 ± 1	67 ± 1	1.8 (−1.8 to 5.3)	70 ± 1	2.5 (−1.1 to 6.1)

*Note:* Data are presented as mean ± SD or number (%). Mean altitude and supplemental oxygen therapy (SOT) effects are expressed as mean differences with 95% confidence intervals. #mean time in bed over all patients assessed under each condition. Cerebral oxygen desaturation events were defined as decreases in cerebral tissue oxygenation (CTO) of at least 4% lasting ≥ 10 s. Severe cerebral desaturation events were defined as CTO decreases ≥ 13% lasting ≥ 10 s. Arterial oxygen desaturation events were defined as decreases in peripheral oxygen saturation (SpO_2_) of at least 3% lasting ≥ 10 s.

Abbreviations: aODI, arterial oxygen desaturation index; cODI, cerebral oxygen desaturation index; CTO, cerebral tissue oxygenation; SpO_2_, peripheral oxygen saturation; SOT, supplemental oxygen therapy.

### 3.2 SOT versus Room Air

**Figure 3:**
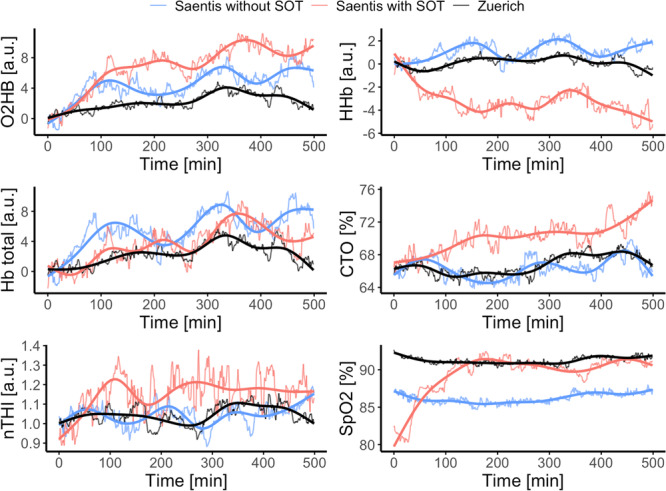
Time course of NIRS‐derived parameters and SpO_2_ during overnight monitoring at low altitude (black), high altitude without supplemental oxygen therapy (SOT) (blue), and high altitude with SOT (red). Data represent one‐second mean values across all patients. Parameters shown are oxygenated hemoglobin (O_2_Hb), deoxygenated hemoglobin (HHb), total hemoglobin (Hb total), cerebral tissue oxygenation (CTO), normalized tissue hemoglobin index (nTHI), and peripheral oxygen saturation (SpO_2_). Bold lines indicate group means; light lines depict individual patient trajectories.

SOT was administered to 8 of 16 patients (50%) at high altitude as a predefined safety measure due to severe nocturnal hypoxemia (SpO_2_ < 80% > 30 min), resulting in significant improvements in SpO_2_ compared with ambient air (Table 2). Nocturnal SpO_2_ increased from 83.6 ± 0.8% under ambient air to 91.1 ± 1.2% with SOT, corresponding to a mean SOT effect of 7.5% (4.9 to 10.0). This improvement in systemic oxygenation was accompanied by a pronounced reduction in arterial oxygen desaturation events. The aODI decreased from 17.8 ± 3.2 events/hour under ambient air to 1.6 ± 4.4 events/hour with SOT (mean SOT effect of −16.2 events/hour; 95%CI −26.2 to −6.3). SOT resulted in no significant changes in CTO and cerebral desaturation events tended to decrease under SOT compared to ambient air at 2500 m (Table 2). Effects of SOT are also illustrated in Figure 3.

Additional cODI‐derived parameters showed a similar pattern. The mean duration of cerebral desaturation events increased under SOT (26.2 ± 3.4 s vs. 20.5 ± 2.5 s under ambient air), while average desaturation depth and the number of severe cerebral desaturation events (CTO decrease ≥ 13%) also differed numerically between conditions; however, none of these comparisons reached statistical significance (Table 2 and Figure 3).

O_2_Hb increased progressively during the night in both groups at high altitude (with and without SOT), with a more pronounced rise in the group receiving SOT. HHb remained constant at low altitude and high altitude with ambient air but decreased gradually in the SOT group. TotHb increased over time in both high altitude groups and remained stable at low altitude. CTO and nTHI showed gradual increases in the SOT group, while remaining stable in the group without SOT.

## 4 Discussion

In this randomized‐controlled crossover study, we investigated nocturnal peripheral and cerebral oxygenation in patients with PVD during the first night at high altitude (2500 m). The key finding was a preserved mean CTO at high altitude. The number of cODI events per hour, duration, and severe desaturations showed a mild but insignificant increase at high altitude despite a marked reduction in peripheral arterial oxygenation, a significant decrease in mean nocturnal SpO_2_ and a pronounced increase in aODI events. These findings are in line with previous studies in PH and healthy individuals, confirming that hypobaric hypoxia during sleep substantially aggravates peripheral oxygen desaturation [6, 14]. This dissociation between peripheral and cerebral oxygenation assessed on ambient air suggests that, at least up to 2500 m, compensatory cerebrovascular mechanisms are able to maintain cerebral oxygen homeostasis despite substantial peripheral oxygen desaturation in this high‐risk PVD‐population, consistent with established concepts of cerebral autoregulation under hypoxic conditions [11, 15, 16].

Comparable results have been reported in hypoxia studies in healthy volunteers, where cerebral oxygenation remained stable despite systemic desaturation, presumably due to increased cerebral perfusion [2, 16]. Our findings extend this concept to patients with PVD, demonstrating that autoregulatory mechanisms to maintain CTO remain functional even in the presence of advanced pulmonary vascular limitation. However, a previous study in lowlanders with moderate‐to‐severe COPD staying overnight at 2590 m demonstrated a pronounced reduction in nocturnal SpO_2_ (−8.8%, 95%CI −10.0 to −7.6), which is comparable to our findings in our PVD‐patients (−6.7%, 95%CI −8.7 to −4.6), although less severe [12]. In contrast to the present PVD‐cohort, patients with COPD also exhibited a significant decrease in CTO, with a mean CTO reduction of −3.6% (95%CI −5.7 to −1.6). In our PVD‐patients, however, mean CTO showed no relevant change at high altitude on ambient air, indicating preserved cerebral oxygenation despite comparable systemic hypoxemia [8, 12].

Nevertheless, half of the PVD‐patients required SOT at 2500 m due to predefined safety criteria of severe nocturnal hypoxemia [17], underscoring the vulnerability of patients with PVD to nocturnal hypoxemia at high altitude [14]. SOT resulted in marked improvements in peripheral oxygenation, with a substantial increase in SpO_2_, reaching levels even exceeding those observed at 470 m, and a pronounced reduction in aODI. These findings are consistent with previous altitude studies in PVD‐patients demonstrating that SOT effectively attenuates altitude‐related hypoxemia and sleep‐disordered breathing [17]. Beyond its pronounced effects on peripheral oxygenation, SOT was associated with insignificantly higher CTO, suggesting a beneficial effect on both peripheral and cerebral oxygenation.

The NIRS‐derived parameters provide additional physiological insights. The observed increases in O_2_Hb and totHb at 2500 m are compatible with an expansion of cerebral blood volume, potentially reflecting cerebral vasodilation, as previously suggested [11, 12]. HHb showed different patterns depending on oxygen therapy. At high altitude without SOT, HHb increased slightly, consistent with enhanced tissue O_2_ extraction in response to reduced arterial O_2_ availability. In contrast, HHb declined modestly in the high‐altitude group with SOT, most likely reflecting higher arterial O_2_ content and improved cerebral oxygen delivery, thereby reducing the need for tissue extraction. Rather than correcting a cerebral oxygen deficit, SOT appears to provide an additional safety margin by reducing systemic desaturation and cerebral oxygen extraction. At low altitude, O_2_Hb, HHb, and totHb remained stable, underscoring that these changes are specific to hypoxic exposure.

The behavior of nTHI is consistent with dynamic adaptations in cerebral hemoglobin concentration at high altitude, suggesting variability in cerebral blood volume and perfusion [16]. The progressive increase in O_2_Hb and totHb at high altitude without a parallel rise in HHb may reflect hypoxia‐related changes in cerebral blood volume and oxygen delivery. While hypocapnia due to hyperventilation is known to induce cerebral vasoconstriction and reduces cerebral blood flow, hypoxia at high altitude has the opposite effect and promoting cerebral vasodilation and increasing perfusion to maintain cerebral oxygen delivery [18]. Whereas PVD‐patients already hyperventilate at low altitude, which would imply a certain cerebral vasoconstriction, it is possible that hypoxia at high altitude opposes this effect. SOT increases arterial oxygen content thereby reducing cerebral blood flow, with net unchanged CTO [15].

Several limitations should be considered. First, the sample size was relatively small, which may have limited statistical power to detect subtle changes in cerebral oxygenation or differences between subgroups. However, the demanding field study in a rare patient collective did not allow to increase sample size. Second, the analysis focused on nocturnal measurements during the first night at altitude, and the findings may not be generalizable to longer exposure periods or higher altitudes. Third, NIRS assesses regional cortical oxygenation and does not provide direct information on global cerebral blood flow or deeper brain structures. Fourth, carbon dioxide levels and direct measures of cerebral perfusion were not assessed, limiting mechanistic insight into cerebrovascular regulation and finally, not all PVD‐patients taken to altitude for an overnight stay had cerebral NIRS measurements available for technical and logistical reasons.

Future studies should evaluate cerebral oxygenation during prolonged altitude exposure and in patients with more advanced disease or lower baseline oxygenation. Combining NIRS with complementary techniques assessing cerebral blood flow and ventilation may further improve understanding of cerebral adaptation to hypoxia in pulmonary vascular disease.

From a clinical perspective, our findings suggest that although patients with PVD experience substantial nocturnal peripheral hypoxemia at 2500 m, CTO is preserved, at least during short‐term exposure on the first night, most likely through effective compensatory mechanisms. Nevertheless, the frequent need for SOT (50% of patients in this cohort) due to predefined safety criteria, which were arbitrarily set according to clinical reasoning and ethical concerns, highlights the importance of careful monitoring and individualized risk assessment when patients with PVD travel to altitude [13].

## 5 Conclusion

In PVD‐patients on ambient air at 2500 m, mean CTO remained preserved despite substantial peripheral oxygen desaturation. These findings indicate that effective compensatory mechanisms can maintain nocturnal cerebral oxygen homeostasis during short‐term exposure to high altitude in stable patients with PVD. In the 50% of patients who received SOT during the night according to predefined safety criteria due to severe hypoxemia, SOT markedly improved peripheral oxygenation but CTO and cODI were unchanged.

## Author Contributions

Conceptualization, Simon R. Schneider, Konrad E. Bloch and Silvia Ulrich; Data curation, C.K and Vera Bissig; Formal analysis, C.K and K. G; Funding acquisition, Silvia Ulrich; Investigation, Simon R. Schneider, Julian Müller, Meret Bauer, Aglaia Forrer, Lea Lüönd, Tanja Ulrich, Arcangelo Carta, Mona Lichtblau, Michael Furian, L.C.M. and Silvia Ulrich; Methodology, Silvia Ulrich; Supervision, Silvia Ulrich; Validation, Mona Lichtblau, Ester I. Schwarz, Konrad E. Bloch and Silvia Ulrich; Visualization, Carolin Kränzle and K.G.; Writing—original draft, Carolin Kränzle; Writing—review and editing, Carolin Kränzle, Vera Bissig, K.G., Michael Furian and Silvia Ulrich All authors have read and agreed to the published version of the manuscript.

## Ethics Statement

The study was approved by the cantonal ethics committee Zurich (KEK Project‐ID 2021‐00243). All participants provided written informed consent.

## Conflicts of Interest

C. Kränzle has nothing to disclose. V. Bissig has nothing to disclose. S. R. Schneider has nothing to disclose. J. Müller has nothing to disclose. K. von Grünigen has nothing to disclose. M. Bauer has nothing to disclose. L. Mayer has nothing to disclose. L. Lüönd has nothing to disclose. T. Ulrich has nothing to disclose. A. Forrer has nothing to disclose. A. Carta has nothing to disclose. E. I. Esther has nothing to disclose. K. E. Bloch has nothing to disclose. M. Lichtblau has nothing to disclose. M. Furian reports grants from the Swiss National Science Foundation and Swiss Lung, outside of the submitted work. S. Ulrich reports grants from Johnson and Johnson SA, Switzerland, during the conduct of the study; and grants from the Swiss National Science Foundation and Zurich Lung, grants and personal fees from Orpha Swiss, and personal fees from Actelion SA and MSD SA, outside the submitted work.

## Supporting information


Supporting File

## Data Availability

The data that support the findings of this study are available on request from the corresponding author. The data are not publicly available due to privacy or ethical restrictions.
